# Antinociceptive Effects of Exogenous and Endogenous Carbon Monoxide in the Nitroglycerin-Induced Migraine Model in Rats

**DOI:** 10.3390/ijms27104346

**Published:** 2026-05-13

**Authors:** Anton Ananev, Karina Gilizhdinova, Dinara Nurmieva, Olga Yakovleva, Kseniia Shaidullova, Guzel Sitdikova

**Affiliations:** Department of Human and Animal Physiology, Institute of Fundamental Medicine and Biology, Kazan Federal University, 420008 Kazan, Russia; anton990124@mail.ru (A.A.); libera350@gmail.com (K.G.); din.nurmieva@yandex.ru (D.N.); a-olay@yandex.ru (O.Y.); k.s.koroleva@yandex.ru (K.S.)

**Keywords:** migraine, trigeminal system, carbon monoxide, nitroglycerin, heme-oxygenase 1, sensitization, mechanical allodynia, photophobia, CGRP, mast cell

## Abstract

Migraines are a common neurological disorder that significantly reduces quality of life. The sensitization of trigeminal afferents is a key factor in the development of the pain syndrome associated with migraine. Carbon monoxide (CO) is produced endogenously by heme oxygenase (HO), widely expressed in structures involved in pain processing. In our study, the role of CO in an acute and chronic nitroglycerin (NTG)-induced rat migraine model was investigated using behavioral, electrophysiological, biochemical and histological methods. The repeated administration of a CO donor (CORM-2) or an HO-1 inducer (CoPP) decreased mechanical hypersensitivity and photophobia of rats in the NTG-induced migraine model. Additionally, CORM-2 and CoPP prevented an increase in trigeminal afferent excitability, which was evaluated by the frequency of action potentials in response to KCl application. Preliminary CORM-2 or CoPP injections promoted mast cell stability in the meninges and prevented NTG-induced CGRP elevation in blood plasma. Our results suggest that exogenously or endogenously produced CO has a protective potential in preventing inflammation and the sensitization of peripheral trigeminal afferents, the activity of which underlies the occurrence of pain in migraine. This could contribute to the development of new approaches for migraine prevention.

## 1. Introduction

Migraine is a common neurological disorder that leads to decreased quality of life, loss of productivity, and disability, affecting 14–15% of the adult population worldwide [1]. Despite significant progress in migraine research and new treatments, fundamental elements of the signaling pathways that induce migraine remain unexplored, and the development of more effective and safer pharmacological treatments is urgently needed [2].

The trigeminovascular system, consisting of the meningeal blood vessels, innervating them with trigeminal afferents, mast cells, and other immune cells, plays a key role in the generation and conduction of nociceptive signaling and is involved in both peripheral and central sensitization, characteristic of patients with chronic migraine [3,4,5]. The calcitonin gene-related peptide (CGRP) released from C-type afferents causes vasodilation of peripheral and cerebral blood vessels [6], which promotes neurogenic inflammation in the dura mater through protein extravasation, release of ATP, and other pro-inflammatory compounds, nitric oxide (NO), all of which play a key role in migraine. Activation of CGRP receptors, expressed mainly on A-delta afferent fibers, induces upregulation of ion channels and promotes nociceptive signaling and hyperexcitability of sensory afferents, which underlie sensitization [7,8,9]. In addition, degranulation of mast cells in the dura mater promotes the release of pro-inflammatory mediators (such as serotonin, prostaglandins, cytokines, etc.), which further provoke nociceptive sensitization of trigeminal nerve afferents [10,11,12,13,14,15,16]. Indeed, elevated levels of CGRP were demonstrated in the plasma of patients with chronic migraine [17].

Carbon monoxide (CO) is a toxic gas that, along with two other members of the gasotransmitter family, NO and hydrogen sulfide (H_2_S), can modulate physiological functions [18]. It is formed endogenously at micromolar concentrations during the breakdown of heme by the enzyme hemoxygenase (HO) into CO, Fe^2+^, and biliverdin, which is then converted to bilirubin [19]. Hemoxygenases occur as two isoforms: inducible HO-1 and constitutive HO-2. HO-1 is present in all tissues and is activated by a number of stress factors, including oxidative stress, which underlies the development of various pathological conditions [20]. HO-2 is widely expressed in the central nervous system, including the cerebral cortex, thalamus, hypothalamus, and brainstem, as well as in the peripheral nervous system: in the trigeminal ganglion, sphenopalatine, superior cervical, and dorsal root ganglion (DRG) [7,21]. These structures are critically important for the pain-sensing system. HO-2 is expressed in the endothelial cells and smooth muscle cells of the cerebral vessels [7,21]. In the trigeminal ganglion, HO-2 has been found in approximately 60% of neurons, and approximately 40% of these neurons also contain CGRP, an established trigger of migraines [22,23].

It is well-known that CO at toxic concentrations induces severe headache, which has been proposed to be mediated by hypoxia [24,25]. Furthermore, exposure to toxic levels of CO is associated with oxidative stress and the generation of reactive oxygen species. This can lead to neurological disorders, such as migraine-like seizures [26]. Whereas the roles of NO and H_2_S in the mechanisms of migraine were demonstrated in experimental models [15,27,28,29,30], the effects of CO on the development of the nociceptive process related to headache in migraine were not investigated.

Injections of nitroglycerin (NTG), an NO donor, into both laboratory animals and humans represent a reliable and reproducible method for modeling both episodic migraine attacks and chronic migraine [27,31,32,33]. HO-2, which is widely expressed in the nucleus of the trigeminal nerve of the spinal cord, functions in conjunction with a marker for all isoforms of NO synthase [34,35]. CO and NO jointly regulate vascular tone. Both substances share a vasodilatory effect by binding to the soluble guanylate cyclase (sGC) and increasing cyclic guanosine monophosphate (cGMP) [36,37]. Experimental studies have shown that CO can modulate NOS expression in a dual manner, depending on the biological concentration, and that NO upregulates HO-1 in smooth muscle and endothelial cells [38,39,40].

Experimental data from recent decades indicate that CO is involved in the nociceptive process. Back in the 90s of the 20th century, it was revealed that intrathecal administration of a non-specific HO inhibitor, zinc protoporphyrin, decreased mechanical allodynia in the hind paw of rats in an inflammatory pain model [41,42]. Later, it was found that HO inhibition reduced nociception in inflammatory, neuropathic, and postoperative pain models mainly through central rather than peripheral mechanisms [43,44,45,46]. These results were supported by studies using recombinant mice with a knock-out of HO-2 (HO-2-KO), in which pain-associated behavior was lower in the inflammatory pain model than in wild-type mice [45,46,47,48]. On the other hand, numerous studies have demonstrated that intraperitoneal, intrathecal, and/or oral administration of CO donors—carbon monoxide-releasing molecules tricarbonyldichlororuthenium (CORMs) such as CORM-2 or CORM-3 or HO-1 inducer—CoPP inhibits acute thermal pain [49], inflammatory pain [50,51], and visceral pain [51,52,53,54,55,56]. In a model of sciatic nerve injury in mice, administration of CORMs and CoPP significantly reduced mechanical and thermal hypersensitivity and increased expression of HO-1 in the DRG and spinal cord.

Because CORM-2 requires iNOS to amplify the HO-1 expression, it was suggested that NO is a key factor in the positive feedback loop that regulates the HO-1 overexpression in neuropathic pain. Along with reduced behavioral response to pain, CORM-2 also decreased activation of spinal microglia and astroglia and expression of matrix metalloproteinase-9 (MMP-9), pro-nociceptive interleukins (IL-1β, IL-18, and IL-6) in the dorsal L4-L6 segments of spinal cord and DRG induced by chronic constriction injury [57]. It was suggested that an increased HO-1 activity is associated with pronounced anti-inflammatory and analgesic effects in acute and chronic pain [21,50,54,55,58,59,60,61,62,63,64,65], whereas the HO-2 overexpression may have a pronociceptive effect [45,66,67,68].

In this study, we analyzed the effects of exogenous CO administration using the CO donor, CORM-2 and the HO-1 inducer, CoPP, on the development of mechanical allodynia and photophobia in the NTG-induced migraine model in rats. In addition, we assessed the excitability of trigeminal afferents using an electrophysiological approach in rats after NTG administration. We also determined the percentage of mast cell degranulation in dura mater and levels of CGRP in rats with the NTG-induced migraine model.

## 2. Results

### 2.1. Effect of CORM II and CoPP on Mechanical Sensitivity in Nitroglycerin-Induced Migraine Model

**Figure 1:**
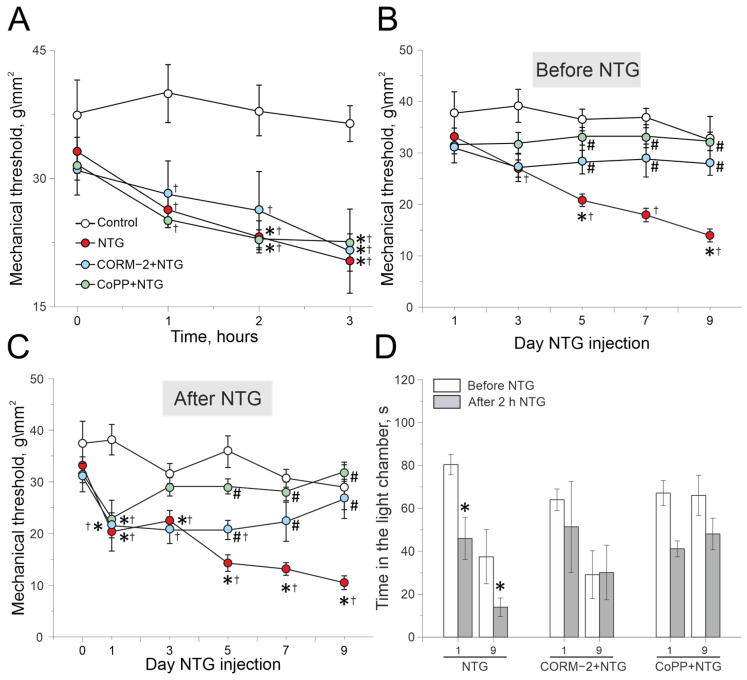
Mechanical thresholds in a nitroglycerin-induced episodic and chronic migraine model. (**A**) Mechanical threshold during three hours after single NTG administration; (**B**) pre-injectional and (**C**) post-injectional thresholds during chronic NTG administration (five injection every second day for 9 days) in the control (white circle; n = 8), NTG (red circle; n = 15), of CORM-2 + NTG (10 mg/kg, blue circle; n = 8), CoPP + NTG (1 mg/kg; green circle; n = 10) groups; (**D**) time spent in light chamber before (white columns) and 2 h after (gray columns) of NTG administration on 1st and 9th days in control (n = 8), NTG (n = 15), CORM-2 + NTG (10 mg/kg, n = 8), CoPP + NTG (1 mg/kg; n = 10) groups; * *p* < 0.05 compared to initial value; # *p* < 0.05 compared to the NTG group; ^†^ *p* < 0.05 compared to the control group.

In the control group, mechanical thresholds were measured after saline injection, and did not change for 3 h (n = 8; Figure 1A). The initial value was 37.5 ± 4.1 g/mm^2^, one hour after injection—39.9 ± 3.4 g/mm^2^ and remained stable 2 and 3 h later (38 ± 2.9 g/mm^2^ and 36.4 ± 2.1 g/mm^2^, respectively). In the NTG group, the mechanical threshold significantly decreased after two hours of NTG administration, compared to the initial level (33.1 ± 1.6 vs. 23.1 ± 1.8 g/mm^2^, n = 15; *p* < 0.001; Fridman ANOVA with post hoc Dunn’s test; Figure 1A), and by the third hour after injection, the mechanical threshold was 20.3 ±1.1 g/mm^2^. Preliminary (1 day before) intraperitoneal administration of the CO donor, CORM-2 (10 mg/kg), and the HO-1 inducer, CoPP (1 mg/kg), did not affect the dynamics of mechanical thresholds after a single NTG administration compared with the NTG group (Figure 1A). In the CORM-2 + NTG group, the mechanical thresholds significantly decreased by 3 h after NTG injections from 30.9 ± 2.8 g/mm^2^ to 21.5 ± 4.9 g/mm^2^ (*p* = 0.04; n = 8; Fridman ANOVA with post hoc Dunn’s test; Figure 1A). In the CoPP + NTG group, mechanical thresholds significantly decreased by two hours after NTG injection, from 31.6 ± 1.8 g/mm^2^ to 22.9 ± 1.1 g/mm^2^ (*p* = 0.008; Fridman ANOVA with post hoc Dunn’s test; n = 10), and after 3 h it was 22.6 ± 0.9 g/mm^2^ (*p* = 0.004; Fridman ANOVA with post hoc Dunn’s test; n = 10; Figure 1A).

Next, mechanical thresholds were assessed before and 2 h after repeated NTG administration (days 3, 5, 7, and 9). In the control group (n = 8), pre-injectional mechanical thresholds did not change during chronic NaCl injections (Figure 1B, Table 1). In the NTG group (n = 15), the initial mechanical threshold was 33.1 ± 1.6 g/mm^2^ and significantly decreased before the third NTG injection (5th day) to 20.8 ± 1.2 g/mm^2^ (*p* = 0.006; Fridman ANOVA with post hoc Dunn’s test); on the day 9, it was 13.9 ± 1.2 g/mm^2^ (*p* = 0.001; Fridman ANOVA with post hoc Dunn’s test; Figure 1B). In the CORM-2 + NTG (n = 8) and CoPP + NTG groups (n = 10), pre-injection mechanical thresholds did not change significantly compared to the initial values (Figure 1B; Table 1).

Next, we analyzed the post-injection mechanical thresholds 3 h after NTG administration during chronic NTG administration.

In the control group (n = 8), five saline injections did not significantly change mechanical thresholds (Figure 1C; Table 2). In the NTG group (n = 15), after the first NTG injection, the mechanical thresholds decreased from 33.1 ± 1.6 g/mm^2^ to 20.3 ± 1.1 g/mm^2^ (*p* = 0.02; Friedman ANOVA with post hoc Dunn’s test; Figure 1C); a further decrease was observed after the third NTG injection (Figure 1C; Table 2). In the CORM-2 + NTG (n = 8) and CoPP + NTG groups (n = 10), the first NTG injection significantly decreased mechanical thresholds, as shown previously (Figure 1C). In the CORM-2 + NTG group, mechanical thresholds after 2nd, 3rd,4th NTG injections did not change further and returned to the initial levels after the fifth NTG injection (Figure 1C, Table 2). In the CoPP + NTG group, the mechanical threshold after the second injection returned to the initial level (Figure 1C, Table 2).

### 2.2. Effects of CORM-2 and CoPP Administration on NTG-Induced Photophobic Behavior in the Light–Dark Transition Test

Photophobia is a specific behavioral sign of a migraine. In the control group (n = 8), animals spent 85.5 ± 6.5 s and 77.2 ± 21.1 s in the light chamber before and after the 1st day of NaCl injection, respectively. At the 5th day, the time spent in the light chamber was 76.3 ± 24.4 s and 74.2 ± 16.8 s before and after NaCl injection, respectively, indicating a lack of photophobic behavior.

In the NTG group (n = 10), animals spent 80.4 ± 4.7 s in the light chamber before, and 45.9 ± 9.7 s (*p* = 0.02; Wilcoxon Signed Ranks Test) after 1st NTG injection (Figure 1D). At the 9th day of experiment, animals spent 37.5 ± 12.6 s in the light chamber before and 13.9 ± 4.3 s after NTG injection (*p* = 0.01; Wilcoxon Signed Ranks Test; Figure 1D), which indicates the development of photophobia. In the CORM-2 + NTG group (n = 8), on the 1st day, animals spent 63.9 ± 5.1 s in the light chamber before, and 51.3 ± 21.1 s after 2 h of NTG injection (*p* = 0.67; Wilcoxon Signed Ranks Test; Figure 1D). On the 9th day before NTG injection, animals spent 29.1 ± 11.1 s in the light chamber (*p* = 0.06 U test compared with the first day) and 30.1 ± 12.7 s (*p* = 0.79; Wilcoxon Signed Ranks Test) 2 h after the injection. In the CoPP + NTG group (n = 10), at the 1st day of NTG administration, animals spent 67.1 ± 5.8 s in the light chamber before, and 41.1 ± 3.68 s after the injection (*p* = 0.10; Wilcoxon Signed Ranks Test; Figure 1D). On the 9th day, animals spent 66.1 ± 9.3 s in the light chamber before and 48.1 ± 7.3 s (*p* = 0.58; Wilcoxon Signed Ranks Test; Figure 1D) after NTG injection.

### 2.3. Excitability of Trigeminal Afferents of Rats in the Model of NTG-Induced Chronic Migraine

**Figure 2:**
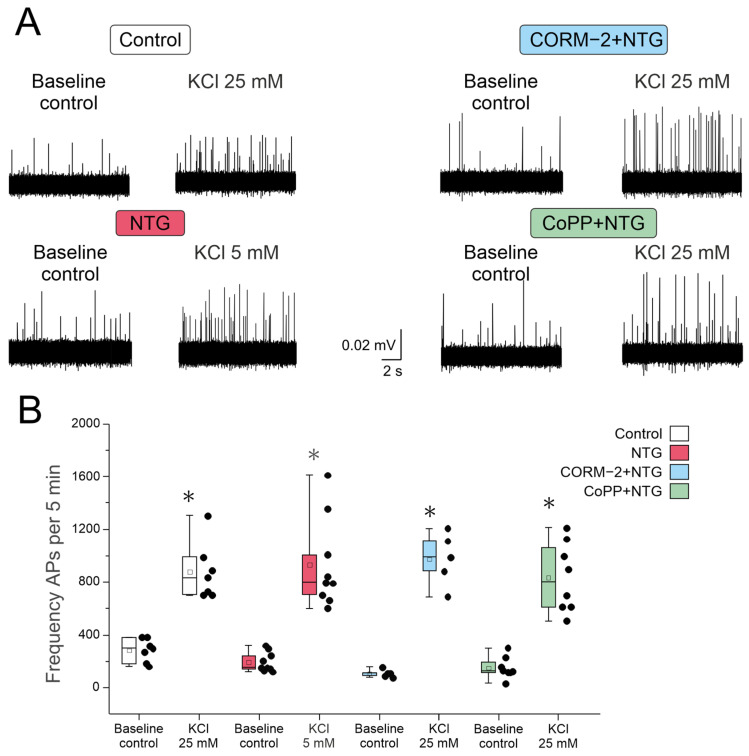
Electrical activity of the trigeminal nerve in response to KCl application. (**A**) The original traces of APs recordings in the trigeminal nerve of rats from the control, NTG, CORM-2 + NTG and CoPP + NTG groups before and after application of KCl; (**B**) box chart of mean number of APs per 5 min in the trigeminal nerve before and after application of KCl. In the control group, the frequency of APs significantly increased at 25 mM KCl (n = 7); in the NTG group, at 5 mM KCl (n = 9). In the CORM-2 + NTG (n = 5) and CoPP + NTG (n = 8) groups, the frequency of APs significantly increased at 25 mM KCl. Data are represented in the box mode (25%,75%), the line is the median, the white square in a box is the mean; whiskers are min and max; black dots—original data. * *p* < 0.05 compared to baseline control (Fridman ANOVA with post hoc Dunn’s test).

To analyze the excitability of trigeminal afferents, a high potassium-containing solution was applied to the receptive area of the dura mater, and the frequency of action potentials (APs) was recorded (Figure 2A).

KCl was applied in concentrations of 5 mM, 10 mM, 25 mM and 50 mM. In rats from the control group, the minimum concentration of KCl that significantly increased the frequency of APs was 25 mM (from 281.4 ± 87.4 APs per 5 min to 872.8 ± 215.1 APs per 5 min; n = 7; Fridman ANOVA with post hoc Dunn’s test *p* = 0.0038; Figure 2A,B; Table 3). A further elevation of KCl concentration to 50 mM decreased trigeminal nerve spiking, evidently due to inactivation of Na^+^ channels. In the NTG group, the baseline frequency of APs was 193.6 ± 24.6 APs per 5 min (n = 9; Figure 2B; Table 3), and 5 mM KCl significantly increased the frequency of APs up to 924.4 ± 112.7 APs per 5 min (n = 9; Fridman ANOVA with post hoc Dunn’s test *p* = 0.001; Figure 2B; Table 3), indicating higher sensitivity to depolarization of trigeminal afferents.

In the CORM-2 + NTG group, AP frequency increased after application of KCl in the concentration of 25 mM similar to the control group from 109.2 ± 13.6 APs per 5 min (n = 5; Figure 2B; Table 3) to 976.1± 89.7 APs per 5 min (n = 5; Fridman ANOVA with post hoc Dunn’s test *p* = 0.001; Figure 2B; Table 3). Similar results were obtained in the CoPP + NTG group. The baseline frequency was 148.8 ± 28.5 APs per 5 min (n = 8; Figure 2B; Table 3), and 25 mM KCl significantly increased the frequency up to 832.1± 92.8 APs per 5 min (n = 8; Fridman ANOVA with post hoc Dunn’s test *p* < 0.001; Figure 2B; Table 3).

### 2.4. Effect of Chronic Administration of NTG on Mast Cell Degranulation in Rats’ Meninges

**Figure 3:**
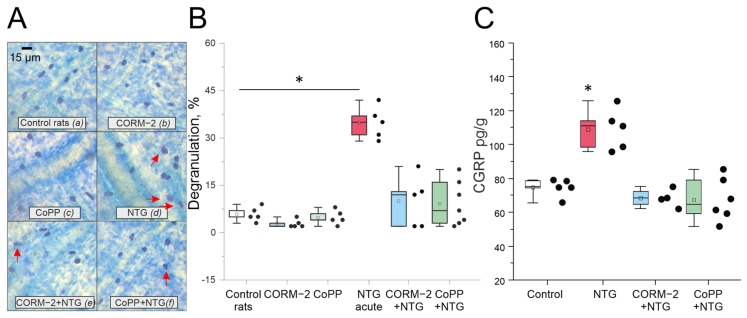
Mast cell degranulation in rat meninges and CGRP level in plasma. (**A**) Toluidine blue staining of the meninges in control (**a**), CORM-2 (**b**), CoPP (**c**), NTG (**d**), CORM-2 + NTG (**e**) and CoPP + NTG (**f**) groups; Red arrow points to degranulated cells. (**B**) the percentage of degranulated mast cells in control, CORM-2, CoPP, NTG, CORM-2 + NTG and CoPP + NTG groups. (**C**) CGRP level in the blood of control, NTG, CORM-2 + NTG and CoPP + NTG groups. Data are represented in the box mode (25%, 75%), the line is the median, the white square in a box is the mean; whiskers are min and max; black dots–original data. * *p* < 0.05 compared to control group (Mann–Whitney U test).

In the control group of rats (n = 5), 5.8 ± 1.1% of meningeal mast cells were degranulated. Administration of CORM-2 (10 mg/kg, n = 5) or CoPP (1 mg/kg, n = 5) did not change mast cells’ degranulation rate: 3.4 ± 0.7% and 4.8 ± 1.1%, respectively (Figure 3A,B). Acute NTG administration increased the number of degranulated mast cells up to 34.4 ± 2.5%, n = 5; *p* = 0.012, U-test, Figure 3B). In the CORM-2 + NTG (n = 7) and CoPP + NTG (n = 7) groups, degranulation rate was significantly lower: 9.3 ± 2.5%, *p* = 0.005, U-test, and 9.1 ± 2.6%, *p* = 0.005, U-test, respectively.

### 2.5. Evaluation of CGRP Level in Blood Plasma of Rats in the Chronic Migraine Model

In the control group (n = 5), before NTG administration, the CGRP level in blood plasma was 73.8 ± 3.1 pg/mL, and after chronic NTG administration, it increased up to 108.9 ± 5.4 pg/mL (*p* = 0.01; Figure 3C). In the CORM-2 + NTG group (n = 5), after chronic NTG administration, the CGRP level was 68.4 ± 2.6 pg/mL (n = 5, *p* = 0.01 compared to the control group; Figure 3C). Similarly, in the CoPP + NTG group (n = 5), the CGRP level did not change after five NTG injections and was 67.4 ± 5.1 pg/mL (n = 5, *p* = 0.01 compared to the control group; Figure 3C).

## 3. Discussion

The present work demonstrates for the first time that repeated intraperitoneal administration of CORM-2 or CoPP prevented the development of mechanical allodynia and photophobia in rats with a chronic NTG-induced migraine model. We have shown that an increase in endogenous CO levels prevented the increase in excitability of trigeminal afferents, whose activity underlies the generation and propagation of nociceptive signaling. Moreover, CORM-2 or CoPP treatment prevented mast cell degranulation and the increase in plasma CGRP in blood plasma in the NTG-induced migraine model.

CO is a gas well-known for its high toxicity, and CO poisoning is accompanied by bilateral headache, nausea, and vomiting [48]. At the same time, CO is generated endogenously in micromolar concentrations when heme is destroyed by the enzyme HO, which exists in two isoforms. HO-2 is widely expressed in both the central and peripheral nervous systems, including key structures involved in pain processing [7,21]. Similar to NO, CO causes vasodilation via activation of calcium-activated potassium channels (K_Ca_-channels) and the cGMP/protein kinase G-signaling pathway [49,57,66,69,70,71], which is also involved in migraine pathophysiology [27,28,72]. Indeed, our previous data demonstrated an increase in nociceptive activity of trigeminal afferents in response to acute CO application, which was prevented by the GC inhibitor [73]. In experimental models of acute and chronic pain, overexpression of the HO-2 isoform has a pronociceptive effect [45,66,67,68], whereas overexpression of the HO-1 isoform is associated with potent anti-inflammatory and antinociceptive action [21,50,54,55,58,59,60,61,62,63,64,65].

HO-1 inducer compounds, such as CoPP, or CO donor—CORM-2, mimic the biological effects of CO, generated by HO-1, including the suppression of inflammation, acute pain, and neuropathic pain resulting from chronic compression of the sciatic nerve [50,54,61,66]. However, the CO involvement in the nociceptive process in migraine headache remains unclear.

In our study, single or repeated NTG administration was used to model episodic and chronic migraines in rodents [16,27,33,74,75,76,77,78]. Previous studies demonstrated that even a single injection of NTG at a dose of 10 mg/kg in rats resulted in sensitization of the trigeminal ganglion neurons and neurons of the trigeminal nucleus caudalis (TNC), which can explain the development of allodynia [27,31,33,79,80]. Chronic injections of NTG increase the number of neurons expressing nNOS and CGRP in the trigeminal ganglion and TNC regions, as well as expression of the neuronal activity marker–c-fos in the TNC area [81,82,83,84,85,86]. These changes reflect central sensitization, which can explain basal allodynia and photophobia that developed during repeated NTG administration, and correspond to migraine-like symptoms. At the same time, repeated administration of CORM-2 or CoPP prevented the decrease in pre-injectional mechanical thresholds. Post-injectional thresholds returned to the initial level after the second NTG injection in the case of CoPP, and CORM-2 prevented further decrease in mechanical threshold in response to NTG administration.

Photophobia in the NTG model is closely related to the sensitization of peripheral and central neurons of the trigeminovascular system [87,88,89,90]. Increased activity of nociceptive trigeminal neurons elevates transmission of pain signals to the brainstem nuclei, including the TNC. The central sensitization of these structures contributes to the fact that sensory stimuli that are not normally painful (for example, light) begin to be perceived as uncomfortable or painful. The convergence of nociceptive and light signals in the posterior thalamus and their subsequent effects on the visual and somatosensory cortex have contributed to the neuroanatomical basis of photophobia [91,92]. The time the animal spent in the light chamber was reduced even after the first NTG injection and further decreased before and after the fifth NTG administration. Repeated administration of CORM-2 prevented changes in total time spent in the light chamber before and after the first and fifth NTG injections. Protective effects of CoPP were observed only after the fifth injection of NTG.

Previously, it was demonstrated that acute and chronic administration of NTG induced hypersensitivity and photophobia, which were associated with a considerable increase in plasma levels of CGRP and pituitary adenylate cyclase-activating peptide (PACAP) [93,94,95], which are involved in the peripheral and central sensitization [96,97]. NTG stimulates the expression of calcium/calmodulin-dependent protein kinase II alpha (CamKIIα) and PACAP receptors—vasoactive intestinal polypeptide type 1 receptors (VPAC1) in the TNC and in the C1-C2 segments of the spinal cord [98]. In addition, NTG decreases serotonin levels in the brainstem with elevation of 5-HT transporter expression [99,100]. At the same time, CO competes with NO by binding with heme and moderately activates sGC, suppresses iNOS [101], and reduces 5-HT synthesis [102]. Thus, CO can both potentiate NTG-dependent cGMP signaling and limit it through NOS inhibition. There is no direct evidence on the effect of CO on 5-HT transporter or VPAC1, which represents an area for future research.

The expression of nNOS and iNOS was shown to be upregulated in the spinal cord and dorsal root ganglia in models of neuropathic pain [66,103,104,105,106]. Allodynia and hyperalgesia induced by nerve injury are significantly diminished or absent in nNOS- and iNOS-deficient animals [66,106,107] or are reversed by the administration of selective NOS, cGMP, and protein kinase G inhibitors [103,105,108]. One of the possible mechanisms of the antinociceptive effects of CO donor and HO-1 inducer in the migraine model is the prevention of iNOS and nNOS upregulation. Similarly, in a model of neuropathic pain induced by sciatic nerve injury, systemic administration of HO-1 inducer reduced thermal and mechanical hyperalgesia and prevented increased expression of iNOS and nNOS in microglia in the spinal cord [66]. At the same time, the systemic administration of CORM-2 or CoPP normalizes nNOS expression induced by peripheral inflammation in the dorsal root ganglia and locus coeruleus [55,56].

The NTG-induced migraine model is considered to be mediated by sensitization of the peripheral and central parts of the trigeminal system [109]. We therefore analyzed the excitability of trigeminal afferents, innervating the middle meningeal artery, using a rat hemiskull ex vivo preparation. KCl at increasing concentrations was used to induce membrane depolarization, followed by an increase in the frequency of AP generation. The effects of KCl are not dependent on specific ion channel or receptor activation, providing us with general information about the membrane state. Moreover, meningeal elevation of KCl was suggested to activate dural afferents during cortical spreading depression.

In preparations of rats with a chronic migraine model (NTG group), the excitability of meningeal afferents was higher compared to the control group, and lower KCl concentrations (5 mM) induced an elevation of AP frequency. Excitability of trigeminal afferents depends on the activity/expression of ion channels involved in maintaining the resting membrane potential and AP generation, such as two-pore potassium channels, voltage-gated sodium, calcium, and potassium channels, and receptor channels specifically involved in nociceptive activity–TRPV1, TRPA1, P2X3, 5HT3 (pain transducers). Indeed, in our previous study, an increased response of trigeminal afferents to capsaicin, a TRPV1 receptor agonist, was shown in rats with chronic NTG administration [27].

Moreover, in rodents, administration of NTG increases the expression of PACAP in TNC and CGRP in the trigeminal ganglion [81,82,110] along with elevation of CGRP levels in blood plasma [28,81,82,83], which was also shown in patients with chronic migraine [111,112]. Release of CGRP from the peripheral C-type terminals initiates a cascade of events that sensitize A-delta afferents. In the trigeminal ganglion, CGRP interacts with neurons and satellite glial cells to promote peripheral sensitization and can drive central sensitization of the second-order neurons [23]. In our study, CGRP level was elevated in the plasma of rats with a chronic NTG model, whereas preliminary administration of CORM-2 and CoPP prevented this effect. Alongside, we did not observe sensitization of trigeminal afferents in these animals, where an increase in AP frequency was only observed in response to 25 mM KCl, similarly to the control group. Ion channels may be potential targets for CO and/or CORM and may mediate their pro- or antinociceptive action. L-type calcium channels can be inhibited or activated through interaction with CO [113]. Two-pore potassium channels (K_2_Ps), specifically the TREK-1 subtype, important in maintaining resting membrane potential and excitability, are activated by CO in low concentrations [114]. Large conductance calcium-activated potassium channels (BK channels) are the most well-known target for CO in smooth muscle cells of arteries, endothelial cells in veins, and the microcirculation of the brain [115]. BK channels are expressed in migraine-associated structures, including cranial arteries, trigeminal ganglia, and spinal trigeminal nucleus [116,117]. These channels play an important role in maintaining vascular tone [118,119] and contribute to nociceptive processing by regulating the amplitude and duration of APs, indicating their key role in neuronal excitability [120].

CGRP in the trigeminal ganglion activates satellite glial cells, which stimulate production of tumor necrosis factor-α (TNF-α) [121], release of interleukin IL-1β and PGE2, which promote neuronal sensitization [122,123,124]. Administration of NTG also activates inflammatory pathways, causing upregulation of pro-inflammatory mediators, including IL-1β and IL-6 in dura mater [125], increases nuclear factor kappa B (NF-κB) and iNOS expression [126]. Altogether, these inflammatory cascades promote mast cell degranulation [125,126], which supports inflammatory reactions and causes direct activation and sensitization of afferents [14,16,109].

Indeed, a single systemic administration of NTG in vivo results in an increase in the number of degranulated mast cells, whereas direct application of NO donors to meningeal structures ex vivo did not induce mast cell degranulation [28,127]. In our study, we found that even a single administration of CORM-2 and CoPP maintains the integrity of mast cells after NTG injection. Similar results were demonstrated in the connective tissue and serosa of rats, where CO and HO-1 inducers prevented mast cell degranulation and histamine release [128,129].

## 4. Materials and Methods

### 4.1. Animals

The experiments were conducted on male Wistar rats (P40-45) in accordance with the European Union Council Directive of 22 September 2010 (2010/63/EEC), and were approved by the local ethics committee of Kazan Federal University (protocol No. 56, dated 19 September 2025). Efforts were made to reduce the number of animals used in experiments.

Nitroglycerin (NTG, Ozon, Moscow, Russia) was injected intraperitoneally (IP) in a concentration of 10 mg/kg (in 0.9% NaCl solution) for modeling episodic and chronic migraine in rats [130,131]. A single injection of NTG is considered a model of episodic migraine. In the model of chronic migraine, NTG was injected 5 times every second day for nine days (days 1, 3, 5, 7, and 9) [130,131].

The study included 4 groups of animals. Animals of the control group obtained injections of 0.9% NaCl (n = 8). Animals of the NTG group obtained 5 injections of NTG every second day for 9 days (n = 15). Animals of the CORM-2 + NTG group obtained 10 mg/kg of CORM-2 every day during the period of NTG injections; on the day of NTG injection, CORM-2 was administered 3 h before NTG n = 8). Animals of the CoPP + NTG group, in addition to NTG, received CoPP (1 mg/kg) every second day, starting 1 day before the first injection of NTG (n = 10).

Mast cell degranulation was assessed in rat meninges after a single injection of NTG (10 mg/kg) with tissue sampling after 3 h (n = 5). CORM-2 (10 mg/kg, n = 5) or CoPP (1 mg/kg, n = 5) were administered 1 day before the NTG injection. CORM-2 or CoPP was dissolved in Dimethyl sulfoxide (DMSO) and then diluted with NaCl to the working concentration. The final concentration of DMSO was 0.01%. Freshly prepared solutions of substances were administered using intraperitoneal injections.

### 4.2. Behavioral Analysis in Rat NTG Migraine Model

Mechanical sensitivity and photophobia were measured before and after NTG administration. In the model of episodic migraine (single NTG administration), mechanical sensitivity was analyzed for 3 h after injection. In the model of chronic migraine, mechanical sensitivity was measured before (pre-injection/basal response) and 2 h after (post-injection response) NTG injections [33]. Photophobia in the light–dark transition test was measured before and two hours after NTG administration at the 1st and 9th day of the experiment in the model of chronic migraine. The test sequence was as follows: von Frey, light–dark box. All behavioral experiments were conducted at the same time and started at approximately 9 a.m.

#### 4.2.1. Von Frey Test

Thresholds of mechanical sensitivity were assessed using a series of calibrated von Frey filaments (Ugo Basile, Gemonio, Italy), ranging from 0.16 g to 4 g, corresponding to a target force of 8.77–40.3 g/mm^2^ according to the up-and-down method [27,31,132]. Thirty minutes before the testing, the rat was placed in a transparent box with a mesh floor. According to the methodology, the tips of von Frey filaments were carefully applied to the sensitive part of the skin between the pads of the hind paws for 1–3 s. All experiments started with a 2 g (37.4 g/mm^2^) filament. A positive response was considered in case of withdrawal, shaking or licking of the paw. In the absence of a reaction, a heavier filament (up) was used after 10–15 s, and in the presence of a reaction, a lighter filament (down) was tested. The final result was the lowest filament value, which the animal responded to in 3 sessions out of 5.

#### 4.2.2. Light–Dark Transition Test

The light–dark transition test was used to assess photophobia in rats [27]. The test apparatus consists of two identical boxes (40 × 20 × 40 cm) connected by a passageway (7 × 7 cm, Open Science, Moscow, Russia). One box was illuminated at a level of 150 lux, while the other was darkened to 1–2 lux. A Sony SSC-G118 (Tokyo, Japan) video camera was used to monitor the experiment. Photophobia testing began with placing a rat in a light chamber. The animal was allowed to freely explore the apparatus for 3 min. The total time spent by the animal in the light chamber was recorded.

### 4.3. Electrophysiological Recording of Action Potentials of Trigeminal Nerve Afferents

The activity of the trigeminal afferents was measured using isolated rat hemiskull preparations with intact dural innervation, as previously described [16,27,29]. The rat hemiskull was placed in a bath, and the trigeminal nerve afferent was carefully isolated from the dura mater under visual control and tightly fixed to a glass electrode. During the entire experiment, the preparation was perfused by an artificial cerebrospinal fluid containing 120 mM NaCl, 2.5 mM KCl, 2 mM CaCl_2_, 1 mM MgCl_2_, 11 mM glucose, 1 mM NaHPO_4_, and 24 mM NaHCO_3_ under constant oxygenation at 95% O_2_/CO_2_, a pH range of 7.2–7.35, and at 20–25 °C. The action potentials (APs) of the trigeminal nerve were captured and transformed into digital data using a DAM 80 amplifier. The data was then processed and visualized on a PC equipped with an NI PCI6221 board (National Instruments, Austin, TX, USA). The software WinEDR v.3.2.7, developed by the University of Strathclyde (Glasgow, UK), was used to visualize the data. The AP frequency was measured and analyzed using the DoClust application in the MATLAB package (MathWorks, Natick, MA, USA; Andrey Zakharov, https://github.com/AndreyZakharovExp (accessed on 1 January 2015)) and OriginPro 8.6 (OriginLab Corporation, Northampton, MA, USA) software. 

### 4.4. Plasma CGRP Concentration

The blood samples were obtained from rats by making an incision in the gum. The samples were subsequently mixed with an anticoagulant in test tubes and subjected to centrifugation at 1500 rpm for 15 min. The resulting supernatant was further stored at a temperature of −40 °C until analysis using the enzyme-linked immunosorbent assay (ELISA) method.

The levels of CGRP were measured using specific ELISA kits designed for rats (CEA876Ra ELISA Kit for Calcitonin Gene-Related Peptide, CGRP, Cloud-Clone Corp., Katy, TX, USA) following the manufacturer’s protocol and the samples were added to the corresponding micro-ELISA strip-plate wells. To each sample, specific antibodies labeled with horseradish peroxidase (HRP) were added for color reaction. The amount of bound HRP conjugates and the color intensity are inversely related to the concentration of CGRP in the sample. We used a microplate spectrophotometer (Multiskan FS, Thermo Fisher Scientific, Waltham, MA, USA) to measure the optical density at a wavelength of 450 nm. The results were analyzed using a calibration curve and expressed in picograms per milliliter (pg/mL).

### 4.5. Toluidine Blue Staining of Meningeal Mast Cells

The histological method of staining the meninges with toluidine blue (Sigma-Aldrich, St. Louis, MO, USA) was used to assess mast cell degranulation [16,27,133]. After incubation, the skulls were rinsed in a phosphate-buffered saline solution with the following composition (mM): 137 NaCl, 2.7 KCl, 10 Na_2_HPO_4_, 1.8 K_2_HPO_4_. The isolated meninges were fixed onto a glass slide and stained with toluidine blue for 10 min. After staining, preparations were washed with distilled water and dehydrated in ethyl alcohol (95–99%). A total of 10 images (20× magnification) were obtained from each preparation, and 100 cells were collected. The degree of degranulation was determined as the % of degranulated cells to the total number of cells [14,16,134].

### 4.6. Data Analysis

Data analysis was performed using the MATLAB software (MathWorks, Natick, USA) and OriginPro 8.6 (OriginLab Corporation, Northampton, MA, USA). The Shapiro–Wilk test was used to assess the normality of the data. All statistical tests were two-tailed, and the nonparametric Mann–Whitney U test was used for comparisons between groups. Fridman ANOVA with post hoc Dunn’s test was used for repeated measures within the same preparation. Statistical significance was set at *p* < 0.05; n indicates the number of animals. All sample sizes were greater than or equal to the suggested sample size calculated in G power for a 0.95 desired power.

## 5. Conclusions

Our study for the first time demonstrated that pre-treatment with the CO donor, CORM-2, and the HO-1 inducer, CoPP, produces protective effects on the behavioral symptoms associated with migraine in the NTG-induced migraine model in rats. This finding was supported by the prevention of peripheral sensitization of trigeminal afferents in the rat migraine model by a preliminary increase in endogenous CO levels. In addition, CORM-2 or CoPP injections prevented the increase in CGRP level and mast cell degranulation in rat meninges. It was proposed that an increase in endogenous CO level may be a promising strategy for developing approaches to migraine prevention.

## Figures and Tables

**Table 1:** Pre-injection mechanical thresholds (g/mm^2^).

Group	1 Day	3 Day	5 Day	7 Day	9 Day
Control (n = 8)	37.5 ± 4.1	38.8 ± 3.1	36.1 ± 2.1	36.5 ± 1.8	32.5 ± 4.2
NTG (n = 15)	33.1 ± 1.6	26.9 ± 1.6 ^†^ (*p* = 0.005)	20.8 ± 1.2 * (*p* = 0.006) ^†^ (*p* < 0.001)	17.9 ± 1.3 ^†^ (*p* < 0.001)	13.9 ± 1.2 * (*p* = 0.001) ^†^ (*p* < 0.001)
CORM-2 + NTG (n = 8)	30.9 ± 2.8	27.1 ± 2.7	28.2 ± 2.2 # (*p* = 0.007)	28.8 ± 3.4 # (*p* = 0.016)	27.9 ± 2.5 # (*p* < 0.001)
CoPP + NTG (n = 10)	31.6 ± 1.8	31.8 ± 2.1	33.2 ± 1.7 # (*p* < 0.001)	33.2 ± 2.2 # (*p* < 0.001)	32.3 ± 1.7 # (*p* < 0.001)

* *p* < 0.05; Fridman ANOVA with post hoc Dunn’s test, within-group comparison; # *p* < 0.05 Mann–Whitney U test, compared to the NTG group; ^†^ *p* < 0.05 compared to the control group.

**Table 2:** Post-injection mechanical thresholds (g/mm^2^).

Group	Initial Value	1 Day	3 Day	5 Day	7 Day	9 Day
Control (n = 8)	37.5 ± 4.1	38.1 ± 2.9	31.5 ± 1.9	35.7 ± 3.1	30.5 ± 1.7	28.9 ± 4.2
NTG (n = 15)	33.1 ± 1.6	20.3 ± 1.1 * (*p* = 0.02) ^†^ (*p* = 0.001)	22.4 ± 1.9 * ^†^ (*p* = 0.004)	14.3 ± 1.61 * (*p* < 0.001) ^†^ (*p* < 0.001)	13.8 ± 1.1 * (*p* < 0.001) ^†^ (*p* < 0.001)	11.5 ± 1.3 * (*p* < 0.001) ^†^ (*p* < 0.001)
CORM-2 + NTG (n = 8)	30.9 ± 2.8	21.5 ± 4.9 * (*p* = 0.04) ^†^ (*p* = 0.04)	20.6 ± 2.5 ^†^ (*p* = 0.005)	20.6 ± 1.8 # (*p* = 0.04) ^†^ (*p* = 0.002)	22.2 ± 3.7 # (*p* = 0.07)	26.6 ± 3.7 # (*p* = 0.001)
CoPP + NTG (n = 10)	31.6 ± 1.8	22.6 ± 0.9 * (*p* = 0.004) ^†^ (*p* < 0.001)	31.8 ± 2.1	29.1 ± 1.4 # (*p* < 0.001)	28.1 ± 1.8 # (*p* < 0.001)	31.9 ± 1.8 # (*p* < 0.001)

* *p* < 0.05; Fridman ANOVA with post hoc Dunn’s test, within-group comparison; # *p* < 0.05 Mann–Whitney U test, compared to the NTG group; ^†^ *p* < 0.05 Mann–Whitney U test compared to the control group.

**Table 3:** Effects of KCl on the electrical activity of the trigeminal nerve.

	Control	NTG	CORM-2 + NTG	CoPP + NTG
Baseline	281.4 ± 87.4	193.6 ±24.6	109.2 ± 13.6	148.8 ± 28.5
KCl 5 mM	326.8 ± 64.5	924.4 ±112.7 * (* *p* = 0.027)	130.8 ± 13.9	218.6 ± 46.7
KCl 10 mM	467.5 ± 230.1	513.8 ± 102.2	472.6 ± 156.5	386.1 ± 51.6
KCl 25 mM	872.8 ± 215.1 * (* *p* = 0.0038)	484.3 ± 105.8	976.1 ± 89.7 (* *p* = 0.001)	832.1 ± 92.8 * (*p* < 0.001)

Mean frequency of the trigeminal nerve AP per 5 min. KCl was applied in increasing concentrations from 5 mM to 50 mM. Significant increase in AP frequency was observed at 25 mM KCl in the control (n = 7), CORM-2 + NTG (n = 5), CoPP + NTG (n = 8) group and at 5 mM in the NTG group (n = 9). * *p* < 0.05 compared to baseline control (Fridman ANOVA with post hoc Dunn’s test).

## Data Availability

The data used to support the findings of this study are available from the corresponding author upon request.

## Abbreviations

The following abbreviations are used in this manuscript:

CO	Carbon monoxide
CORM	Carbon dioxide-releasing molecule
HO	Heme oxygenase
CoPP	Cobalt Protoporphyrin IX
CGRP	Calcitonin gene-related peptide
NTG	Nitroglycerin
NO	Nitric oxide
H2S	Hydrogen sulfide
DRG	Dorsal root ganglion
sGC	Soluble guanylate cyclase
cGMP	Cyclic guanosine monophosphate
NOS	Nitric oxide synthase
DMSO	Dimethyl sulfoxide
TNC	Trigeminal nucleus caudalis
PACAP	Pituitary adenylate cyclase-activating peptide

## References

[1] Steiner T.J., Stovner L.J. (2023). Global Epidemiology of Migraine and Its Implications for Public Health and Health Policy. Nat. Rev. Neurol..

[2] Ferrari M.D., Goadsby P.J., Burstein R., Kurth T., Ayata C., Charles A., Ashina M., van den Maagdenberg A.M.J.M., Dodick D.W. (2022). Migraine. Nat. Rev. Dis. Primer.

[3] Kekere V., Alsayouri K. (2026). Anatomy, Head and Neck, Dura Mater. StatPearls.

[4] Rua R., McGavern D.B. (2018). Advances in Meningeal Immunity. Trends Mol. Med..

[5] Ashina M., Hansen J.M., Do T.P., Melo-Carrillo A., Burstein R., Moskowitz M.A. (2019). Migraine and the Trigeminovascular System-40 Years and Counting. Lancet Neurol..

[6] Durham P.L. (2006). Calcitonin Gene-Related Peptide (CGRP) and Migraine. Headache.

[7] Olesen J., Burstein R., Ashina M., Tfelt-Hansen P. (2009). Origin of Pain in Migraine: Evidence for Peripheral Sensitisation. Lancet Neurol..

[8] Edvinsson L., Uddman R. (2005). Neurobiology in Primary Headaches. Brain Res. Brain Res. Rev..

[9] Russo A.F. (2015). Calcitonin Gene-Related Peptide (CGRP): A New Target for Migraine. Annu. Rev. Pharmacol. Toxicol..

[10] Edvinsson L., Haanes K.A., Warfvinge K. (2019). Does Inflammation Have a Role in Migraine?. Nat. Rev. Neurol..

[11] Suleimanova A., Talanov M., Gafurov O., Gafarov F., Koroleva K., Virenque A., Noe F.M., Mikhailov N., Nistri A., Giniatullin R. (2020). Modeling a Nociceptive Neuro-Immune Synapse Activated by ATP and 5-HT in Meninges: Novel Clues on Transduction of Chemical Signals Into Persistent or Rhythmic Neuronal Firing. Front. Cell. Neurosci..

[12] Biscetti L., Cresta E., Cupini L.M., Calabresi P., Sarchielli P. (2023). The Putative Role of Neuroinflammation in the Complex Pathophysiology of Migraine: From Bench to Bedside. Neurobiol. Dis..

[13] Spekker E., Tanaka M., Szabó Á., Vécsei L. (2021). Neurogenic Inflammation: The Participant in Migraine and Recent Advancements in Translational Research. Biomedicines.

[14] Nurkhametova D.F., Koroleva K.S., Gafurov O.S., Giniatullina R.R., Sitdikova G.F., Giniatullin R.A. (2020). Mast Cell Mediators as Pain Triggers in Migraine: Comparison of Histamine and Serotonin in the Activation of Primary Afferents in the Meninges in Rats. Neurosci. Behav. Physiol..

[15] Koroleva K., Ermakova E., Mustafina A., Giniatullina R., Giniatullin R., Sitdikova G. (2020). Protective Effects of Hydrogen Sulfide Against the ATP-Induced Meningeal Nociception. Front. Cell. Neurosci..

[16] Koroleva K., Gafurov O., Guselnikova V., Nurkhametova D., Giniatullina R., Sitdikova G., Mattila O.S., Lindsberg P.J., Malm T.M., Giniatullin R. (2019). Meningeal Mast Cells Contribute to ATP-Induced Nociceptive Firing in Trigeminal Nerve Terminals: Direct and Indirect Purinergic Mechanisms Triggering Migraine Pain. Front. Cell. Neurosci..

[17] Yamanaka G., Hayashi K., Morishita N., Takeshita M., Ishii C., Suzuki S., Ishimine R., Kasuga A., Nakazawa H., Takamatsu T. (2023). Experimental and Clinical Investigation of Cytokines in Migraine: A Narrative Review. Int. J. Mol. Sci..

[18] Hermann A., Sitdikova G.F., Weiger T.M. (2012). Gasotransmitters: Physiology and Pathophysiology.

[19] Motterlini R., Otterbein L.E. (2010). The Therapeutic Potential of Carbon Monoxide. Nat. Rev. Drug Discov..

[20] Motterlini R., Foresti R. (2017). Biological Signaling by Carbon Monoxide and Carbon Monoxide-Releasing Molecules. Am. J. Physiol. Cell Physiol..

[21] Fan W., Huang F., Wu Z., Zhu X., Li D., He H. (2011). Carbon Monoxide: A Gas That Modulates Nociception. J. Neurosci. Res..

[22] Uddman R., Tajti J., Sundler F., Cardell L.-O. (2004). The Presence of Heme-Oxygenase and Biliverdin Reductase in Human Cranial Ganglia Indicates a Role for Carbon Monoxide in Neural Transmission. Neuro Endocrinol. Lett..

[23] Iyengar S., Johnson K.W., Ossipov M.H., Aurora S.K. (2019). CGRP and the Trigeminal System in Migraine. Headache.

[24] Hampson N.B., Hampson L.A. (2002). Characteristics of Headache Associated with Acute Carbon Monoxide Poisoning. Headache.

[25] Lipton R.B., Mazer C., Newman L.C., Solomon S. (1997). Sumatriptan Relieves Migrainelike Headaches Associated with Carbon Monoxide Exposure. Headache.

[26] Mulay P., Chen C., Krishna V. (2023). Enzyme-Independent Catabolism of Cysteine with Pyridoxal-5’-Phosphate. Sci. Rep..

[27] Koroleva K., Svitko S., Ananev A., Buglinina A., Bogatova K., Yakovleva O., Nurmieva D., Shaidullov I., Sitdikova G. (2023). Effects of Nitric Oxide on the Activity of P2X and TRPV1 Receptors in Rat Meningeal Afferents of the Trigeminal Nerve. Int. J. Mol. Sci..

[28] Koroleva K.S., Svitko S.O., Nurmieva D.A., Gafurov O.S., Buglinina A.D., Sitdikova G.F. (2022). Effects of Nitric Oxide on the Electrical Activity of the Rat Trigeminal Nerve and Mast Cell Morphology. J. Evol. Biochem. Physiol..

[29] Koroleva K., Mustafina A., Yakovlev A., Hermann A., Giniatullin R., Sitdikova G. (2017). Receptor Mechanisms Mediating the Pro-Nociceptive Action of Hydrogen Sulfide in Rat Trigeminal Neurons and Meningeal Afferents. Front. Cell. Neurosci..

[30] Messlinger K., Lennerz J.K., Eberhardt M., Fischer M.J.M. (2012). CGRP and NO in the Trigeminal System: Mechanisms and Role in Headache Generation. Headache.

[31] Gerasimova E., Yakovleva O., Enikeev D., Bogatova K., Hermann A., Giniatullin R., Sitdikova G. (2022). Hyperhomocysteinemia Increases Cortical Excitability and Aggravates Mechanical Hyperalgesia and Anxiety in a Nitroglycerine-Induced Migraine Model in Rats. Biomolecules.

[32] Demartini C., Greco R., Zanaboni A.M., Sances G., De Icco R., Borsook D., Tassorelli C. (2019). Nitroglycerin as a Comparative Experimental Model of Migraine Pain: From Animal to Human and Back. Prog. Neurobiol..

[33] Sureda-Gibert P., Romero-Reyes M., Akerman S. (2022). Nitroglycerin as a Model of Migraine: Clinical and Preclinical Review. Neurobiol. Pain.

[34] Fan W., Dong W., Leng S., Li D., Cheng S., Li C., Qu H., He H. (2008). Expression and Colocalization of NADPH-Diaphorase and Heme Oxygenase-2 in Trigeminal Ganglion and Mesencephalic Trigeminal Nucleus of the Rat. J. Mol. Histol..

[35] Fan W., Huang F., Dong W., Gao Z., Li C., Zhu X., Li D., He H. (2009). Distribution of Heme Oxygenase-2 and NADPH-Diaphorase in the Spinal Trigeminal Nucleus of the Rat. J. Mol. Histol..

[36] Nascimento C.G.O., Branco L.G.S. (2008). Role of the Spinal Cord Heme Oxygenase-Carbon Monoxide-cGMP Pathway in the Nociceptive Response of Rats. Eur. J. Pharmacol..

[37] Carvalho P.G., Branco L.G.S., Panissi C.R.A.L. (2011). Involvement of the Heme Oxygenase-Carbon Monoxide-cGMP Pathway in the Nociception Induced by Acute Painful Stimulus in Rats. Brain Res..

[38] Choi Y.K., Kim Y.-M. (2021). Regulation of Endothelial and Vascular Functions by Carbon Monoxide via Crosstalk with Nitric Oxide. Front. Cardiovasc. Med..

[39] Thorup C., Jones C.L., Gross S.S., Moore L.C., Goligorsky M.S. (1999). Carbon Monoxide Induces Vasodilation and Nitric Oxide Release but Suppresses Endothelial NOS. Am. J. Physiol..

[40] Rose J.J., Wang L., Xu Q., McTiernan C.F., Shiva S., Tejero J., Gladwin M.T. (2017). Carbon Monoxide Poisoning: Pathogenesis, Management, and Future Directions of Therapy. Am. J. Respir. Crit. Care Med..

[41] Meller S.T., Dykstra C.L., Gebhart G.F. (1994). Investigations of the Possible Role for Carbon Monoxide (CO) in Thermal and Mechanical Hyperalgesia in the Rat. Neuroreport.

[42] Yamamoto T., Nozaki-Taguchi N. (1995). Zinc Protoporphyrin IX, an Inhibitor of the Enzyme That Produces Carbon Monoxide, Blocks Spinal Nociceptive Transmission Evoked by Formalin Injection in the Rat. Brain Res..

[43] Li X., Angst M.S., Clark J.D. (2001). A Murine Model of Opioid-Induced Hyperalgesia. Brain Res. Mol. Brain Res..

[44] Li X., Clark J.D. (2002). Spinal Cord Heme Oxygenase Participates in Glutamate-Induced Pain-Related Behaviors. Eur. J. Pharmacol..

[45] Li X., Clark J.D. (2003). Heme Oxygenase Type 2 Participates in the Development of Chronic Inflammatory and Neuropathic Pain. J. Pain.

[46] Li X., Lighthall G., Liang D.-Y., Clark J.D. (2004). Alterations in Spinal Cord Gene Expression after Hindpaw Formalin Injection. J. Neurosci. Res..

[47] Li X., Clark J.D. (2000). Heme Oxygenase Type 2 Plays a Role in Formalin-Induced Nociception. Pain.

[48] Arngrim N., Schytz H.W., Hauge M.K., Ashina M., Olesen J. (2014). Carbon Monoxide May Be an Important Molecule in Migraine and Other Headaches. Cephalalgia Int. J. Headache.

[49] Gou G., Leánez S., Pol O. (2014). The Role of Gaseous Neurotransmitters in the Antinociceptive Effects of Morphine during Acute Thermal Pain. Eur. J. Pharmacol..

[50] Rosa A.O., Egea J., Lorrio S., Rojo A.I., Cuadrado A., López M.G. (2008). Nrf2-Mediated Haeme Oxygenase-1 up-Regulation Induced by Cobalt Protoporphyrin Has Antinociceptive Effects against Inflammatory Pain in the Formalin Test in Mice. Pain.

[51] Hervera A., Gou G., Leánez S., Pol O. (2013). Effects of Treatment with a Carbon Monoxide-Releasing Molecule and a Heme Oxygenase 1 Inducer in the Antinociceptive Effects of Morphine in Different Models of Acute and Chronic Pain in Mice. Psychopharmacology.

[52] Carcolé M., Castany S., Leánez S., Pol O. (2014). Treatment with a Heme Oxygenase 1 Inducer Enhances the Antinociceptive Effects of Μ-Opioid, δ-Opioid, and Cannabinoid 2 Receptors during Inflammatory Pain. J. Pharmacol. Exp. Ther..

[53] Parenti C., Aricò G., Chiechio S., Di Benedetto G., Parenti R., Scoto G.M. (2015). Involvement of the Heme-Oxygenase Pathway in the Antiallodynic and Antihyperalgesic Activity of *Harpagophytum procumbens* in Rats. Molecules.

[54] Ferrándiz M.L., Maicas N., García-Arnandis I., Terencio M.C., Motterlini R., Devesa I., Joosten L.A.B., van den Berg W.B., Alcaraz M.J. (2008). Treatment with a CO-Releasing Molecule (CORM-3) Reduces Joint Inflammation and Erosion in Murine Collagen-Induced Arthritis. Ann. Rheum. Dis..

[55] Negrete R., Hervera A., Leánez S., Pol O. (2014). Treatment with a Carbon Monoxide-Releasing Molecule Inhibits Chronic Inflammatory Pain in Mice: Nitric Oxide Contribution. Psychopharmacology.

[56] Moreno P., Cazuza R.A., Mendes-Gomes J., Díaz A.F., Polo S., Leánez S., Leite-Panissi C.R.A., Pol O. (2019). The Effects of Cobalt Protoporphyrin IX and Tricarbonyldichlororuthenium (II) Dimer Treatments and Its Interaction with Nitric Oxide in the Locus Coeruleus of Mice with Peripheral Inflammation. Int. J. Mol. Sci..

[57] Jurga A.M., Piotrowska A., Makuch W., Przewlocka B., Mika J. (2017). Blockade of P2X4 Receptors Inhibits Neuropathic Pain-Related Behavior by Preventing MMP-9 Activation and, Consequently, Pronociceptive Interleukin Release in a Rat Model. Front. Pharmacol..

[58] Hervera A., Leánez S., Motterlini R., Pol O. (2013). Treatment with Carbon Monoxide-Releasing Molecules and an HO-1 Inducer Enhances the Effects and Expression of µ-Opioid Receptors during Neuropathic Pain. Anesthesiology.

[59] Devesa I., Ferrándiz M.L., Terencio M.C., Joosten L.A.B., van den Berg W.B., Alcaraz M.J. (2005). Influence of Heme Oxygenase 1 Modulation on the Progression of Murine Collagen-Induced Arthritis. Arthritis Rheum..

[60] Sawle P., Foresti R., Mann B.E., Johnson T.R., Green C.J., Motterlini R. (2005). Carbon Monoxide-Releasing Molecules (CO-RMs) Attenuate the Inflammatory Response Elicited by Lipopolysaccharide in RAW264.7 Murine Macrophages. Br. J. Pharmacol..

[61] Guillén M.I., Megías J., Clérigues V., Gomar F., Alcaraz M.J. (2008). The CO-Releasing Molecule CORM-2 Is a Novel Regulator of the Inflammatory Process in Osteoarthritic Chondrocytes. Rheumatology.

[62] De Backer O., Elinck E., Blanckaert B., Leybaert L., Motterlini R., Lefebvre R.A. (2009). Water-Soluble CO-Releasing Molecules Reduce the Development of Postoperative Ileus via Modulation of MAPK/HO-1 Signalling and Reduction of Oxidative Stress. Gut.

[63] Megías J., Guillén M.I., Clérigues V., Rojo A.I., Cuadrado A., Castejón M.A., Gomar F., Alcaraz M.J. (2009). Heme Oxygenase-1 Induction Modulates Microsomal Prostaglandin E Synthase-1 Expression and Prostaglandin E(2) Production in Osteoarthritic Chondrocytes. Biochem. Pharmacol..

[64] Maicas N., Ferrándiz M.L., Devesa I., Motterlini R., Koenders M.I., van den Berg W.B., Alcaraz M.J. (2010). The CO-Releasing Molecule CORM-3 Protects against Articular Degradation in the K/BxN Serum Transfer Arthritis Model. Eur. J. Pharmacol..

[65] Bijjem K.R.V., Padi S.S.V., lal Sharma P. (2013). Pharmacological Activation of Heme Oxygenase (HO)-1/Carbon Monoxide Pathway Prevents the Development of Peripheral Neuropathic Pain in Wistar Rats. Naunyn. Schmiedebergs Arch. Pharmacol..

[66] Hervera A., Leánez S., Negrete R., Motterlini R., Pol O. (2012). Carbon Monoxide Reduces Neuropathic Pain and Spinal Microglial Activation by Inhibiting Nitric Oxide Synthesis in Mice. PLoS ONE.

[67] Gordh T., Sharma H.S., Azizi M., Alm P., Westman J. (2000). Spinal Nerve Lesion Induces Upregulation of Constitutive Isoform of Heme Oxygenase in the Spinal Cord. An Immunohistochemical Investigation in the Rat. Amino Acids.

[68] Li X., Clark J.D. (2000). The Role of Heme Oxygenase in Neuropathic and Incisional Pain. Anesth. Analg..

[69] Svitko S.O., Ananev A.S., Nevsky E.S., Shaidullova K.S., Sitdikova G.F. (2025). Gasotransmitters (NO, H_2_S, CO) as Contributors to the Nociceptive Process in the Trigeminovascular System. Neurochem. J..

[70] Chen Y., Chen H., Xie K., Liu L., Li Y., Yu Y., Wang G. (2015). H_2_ Treatment Attenuated Pain Behavior and Cytokine Release Through the HO-1/CO Pathway in a Rat Model of Neuropathic Pain. Inflammation.

[71] Jurga A.M., Piotrowska A., Starnowska J., Rojewska E., Makuch W., Mika J. (2016). Treatment with a Carbon Monoxide-Releasing Molecule (CORM-2) Inhibits Neuropathic Pain and Enhances Opioid Effectiveness in Rats. Pharmacol. Rep..

[72] Olesen J. (2008). The Role of Nitric Oxide (NO) in Migraine, Tension-Type Headache and Cluster Headache. Pharmacol. Ther..

[73] Ananev A.S., Petrova K.A., Gaifutdinova N.R., Gilizhdinova K.R., Svitko S.O., Shaidullova K.S., Sitdikova G.F. (2025). Effect of Carbon Monoxide on ATP-Evoked Activity in Rat Trigeminal Afferents. Biochem. Mosc. Suppl. Ser. Membr. Cell Biol..

[74] Pradhan A.A., Smith M.L., McGuire B., Tarash I., Evans C.J., Charles A. (2014). Characterization of a Novel Model of Chronic Migraine. Pain.

[75] Kilinc E., Guerrero-Toro C., Zakharov A., Vitale C., Gubert-Olive M., Koroleva K., Timonina A., Luz L.L., Shelukhina I., Giniatullina R. (2017). Serotonergic Mechanisms of Trigeminal Meningeal Nociception: Implications for Migraine Pain. Neuropharmacology.

[76] Levy D., Burstein R., Kainz V., Jakubowski M., Strassman A.M. (2007). Mast Cell Degranulation Activates a Pain Pathway Underlying Migraine Headache. Pain.

[77] Schueler M., Neuhuber W.L., De Col R., Messlinger K. (2014). Innervation of Rat and Human Dura Mater and Pericranial Tissues in the Parieto-Temporal Region by Meningeal Afferents. Headache.

[78] Jacobs B., Dussor G. (2016). Neurovascular Contributions to Migraine: Moving beyond Vasodilation. Neuroscience.

[79] Zhang X., Kainz V., Zhao J., Strassman A.M., Levy D. (2013). Vascular Extracellular Signal-Regulated Kinase Mediates Migraine-Related Sensitization of Meningeal Nociceptors. Ann. Neurol..

[80] Akerman S., Karsan N., Bose P., Hoffmann J.R., Holland P.R., Romero-Reyes M., Goadsby P.J. (2019). Nitroglycerine Triggers Triptan-Responsive Cranial Allodynia and Trigeminal Neuronal Hypersensitivity. Brain J. Neurol..

[81] Dieterle A., Fischer M.J.M., Link A.S., Neuhuber W.L., Messlinger K. (2011). Increase in CGRP- and nNOS-Immunoreactive Neurons in the Rat Trigeminal Ganglion after Infusion of an NO Donor. Cephalalgia Int. J. Headache.

[82] Pardutz A., Krizbai I., Multon S., Vecsei L., Schoenen J. (2000). Systemic Nitroglycerin Increases nNOS Levels in Rat Trigeminal Nucleus Caudalis. Neuroreport.

[83] Tassorelli C., Joseph S.A. (1995). Systemic Nitroglycerin Induces Fos Immunoreactivity in Brainstem and Forebrain Structures of the Rat. Brain Res..

[84] Ramachandran R., Bhatt D.K., Ploug K.B., Hay-Schmidt A., Jansen-Olesen I., Gupta S., Olesen J. (2014). Nitric Oxide Synthase, Calcitonin Gene-Related Peptide and NK-1 Receptor Mechanisms Are Involved in GTN-Induced Neuronal Activation. Cephalalgia Int. J. Headache.

[85] Nagy-Grócz G., Tar L., Bohár Z., Fejes-Szabó A., Laborc K.F., Spekker E., Vécsei L., Párdutz Á. (2016). The Modulatory Effect of Anandamide on Nitroglycerin-Induced Sensitization in the Trigeminal System of the Rat. Cephalalgia Int. J. Headache.

[86] Greco R., Ferrigno A., Demartini C., Zanaboni A., Mangione A.S., Blandini F., Nappi G., Vairetti M., Tassorelli C. (2015). Evaluation of ADMA-DDAH-NOS Axis in Specific Brain Areas Following Nitroglycerin Administration: Study in an Animal Model of Migraine. J. Headache Pain.

[87] Denuelle M., Boulloche N., Payoux P., Fabre N., Trotter Y., Géraud G. (2011). A PET Study of Photophobia during Spontaneous Migraine Attacks. Neurology.

[88] Drummond P.D., Woodhouse A. (1993). Painful Stimulation of the Forehead Increases Photophobia in Migraine Sufferers. Cephalalgia Int. J. Headache.

[89] Kowacs P.A., Piovesan E.J., Werneck L.C., Tatsui C.E., Lange M.C., Ribas L.C., da Silva H.P. (2001). Influence of Intense Light Stimulation on Trigeminal and Cervical Pain Perception Thresholds. Cephalalgia Int. J. Headache.

[90] Moulton E.A., Becerra L., Borsook D. (2009). An fMRI Case Report of Photophobia: Activation of the Trigeminal Nociceptive Pathway. Pain.

[91] Noseda R., Kainz V., Jakubowski M., Gooley J.J., Saper C.B., Digre K., Burstein R. (2010). A Neural Mechanism for Exacerbation of Headache by Light. Nat. Neurosci..

[92] Brennan K.C., Pietrobon D. (2018). A Systems Neuroscience Approach to Migraine. Neuron.

[93] Farajdokht F., Babri S., Karimi P., Alipour M.R., Bughchechi R., Mohaddes G. (2017). Chronic Ghrelin Treatment Reduced Photophobia and Anxiety-like Behaviors in Nitroglycerin- Induced Migraine: Role of Pituitary Adenylate Cyclase-Activating Polypeptide. Eur. J. Neurosci..

[94] Farajdokht F., Mohaddes G., Shanehbandi D., Karimi P., Babri S. (2018). Ghrelin Attenuated Hyperalgesia Induced by Chronic Nitroglycerin: CGRP and TRPV1 as Targets for Migraine Management. Cephalalgia Int. J. Headache.

[95] Farajdokht F., Babri S., Karimi P., Mohaddes G. (2016). Ghrelin Attenuates Hyperalgesia and Light Aversion-Induced by Nitroglycerin in Male Rats. Neurosci. Lett..

[96] Greco R., Demartini C., Francavilla M., Zanaboni A.M., Tassorelli C. (2022). Antagonism of CGRP Receptor: Central and Peripheral Mechanisms and Mediators in an Animal Model of Chronic Migraine. Cells.

[97] Ruby H.A., Sayed R.H., Khattab M.A., Sallam N.A., Kenway S.A. (2024). Fenofibrate Ameliorates Nitroglycerin-Induced Migraine in Rats: Role of CGRP/p-CREB/P2X3 and NGF/PKC/ASIC3 Signaling Pathways. Eur. J. Pharmacol..

[98] Nagy-Grócz G., Spekker E., Körtési T., Laborc K.F., Bohár Z., Fejes-Szabó A., Vécsei L., Párdutz Á. (2025). CamKIIα and VPAC1 Expressions in the Caudal Trigeminal Nucleus of Rats After Systemic Nitroglycerin Treatment: Interaction with Anandamide. Life.

[99] Tassorelli C., Blandini F., Costa A., Preza E., Nappi G. (2002). Nitroglycerin-Induced Activation of Monoaminergic Transmission in the Rat. Cephalalgia Int. J. Headache.

[100] Nagy-Grócz G., Bohár Z., Fejes-Szabó A., Laborc K.F., Spekker E., Tar L., Vécsei L., Párdutz Á. (2017). Nitroglycerin Increases Serotonin Transporter Expression in Rat Spinal Cord but Anandamide Modulated This Effect. J. Chem. Neuroanat..

[101] Magierowska K., Magierowski M., Surmiak M., Adamski J., Mazur-Bialy A.I., Pajdo R., Sliwowski Z., Kwiecien S., Brzozowski T. (2016). The Protective Role of Carbon Monoxide (CO) Produced by Heme Oxygenases and Derived from the CO-Releasing Molecule CORM-2 in the Pathogenesis of Stress-Induced Gastric Lesions: Evidence for Non-Involvement of Nitric Oxide (NO). Int. J. Mol. Sci..

[102] Muraoka M., Hayakawa H., Kagaya A., Kojima T., Yamawaki S. (1998). Effects of Carbon Monoxide Exposure on Serotonergic Neuronal Systems in Rat Brain. Life Sci..

[103] De Alba J., Clayton N.M., Collins S.D., Colthup P., Chessell I., Knowles R.G. (2006). GW274150, a Novel and Highly Selective Inhibitor of the Inducible Isoform of Nitric Oxide Synthase (iNOS), Shows Analgesic Effects in Rat Models of Inflammatory and Neuropathic Pain. Pain.

[104] LaBuda C.J., Koblish M., Tuthill P., Dolle R.E., Little P.J. (2006). Antinociceptive Activity of the Selective iNOS Inhibitor AR-C102222 in Rodent Models of Inflammatory, Neuropathic and Post-Operative Pain. Eur. J. Pain.

[105] Schmidtko A., Gao W., König P., Heine S., Motterlini R., Ruth P., Schlossmann J., Koesling D., Niederberger E., Tegeder I. (2008). cGMP Produced by NO-Sensitive Guanylyl Cyclase Essentially Contributes to Inflammatory and Neuropathic Pain by Using Targets Different from cGMP-Dependent Protein Kinase I. J. Neurosci..

[106] Levy D., Zochodne D.W. (1998). Local Nitric Oxide Synthase Activity in a Model of Neuropathic Pain. Eur. J. Neurosci..

[107] Kuboyama K., Tsuda M., Tsutsui M., Toyohara Y., Tozaki-Saitoh H., Shimokawa H., Yanagihara N., Inoue K. (2011). Reduced Spinal Microglial Activation and Neuropathic Pain after Nerve Injury in Mice Lacking All Three Nitric Oxide Synthases. Mol. Pain.

[108] Hervera A., Negrete R., Leánez S., Martín-Campos J., Pol O. (2010). The Role of Nitric Oxide in the Local Antiallodynic and Antihyperalgesic Effects and Expression of Delta-Opioid and Cannabinoid-2 Receptors during Neuropathic Pain in Mice. J. Pharmacol. Exp. Ther..

[109] Svitko S., Ermakova E., Gilizhdinova K., Bogatova K., Gaifutdinova N., Nurmieva D., Nevsky E., Ananev A., Yakovleva O., Sufianov A. (2025). LPS-Induced Neuroinflammation Increases Serotonin-Evoked Activity of Trigeminal Afferents and Aggravates Mechanical Allodynia and Photophobic Behavior in Rat Migraine Model. Int. J. Mol. Sci..

[110] Tuka B., Helyes Z., Markovics A., Bagoly T., Németh J., Márk L., Brubel R., Reglődi D., Párdutz A., Szolcsányi J. (2012). Peripheral and Central Alterations of Pituitary Adenylate Cyclase Activating Polypeptide-like Immunoreactivity in the Rat in Response to Activation of the Trigeminovascular System. Peptides.

[111] Cernuda-Morollón E., Larrosa D., Ramón C., Vega J., Martínez-Camblor P., Pascual J. (2013). Interictal Increase of CGRP Levels in Peripheral Blood as a Biomarker for Chronic Migraine. Neurology.

[112] Greco R., De Icco R., Demartini C., Zanaboni A.M., Tumelero E., Sances G., Allena M., Tassorelli C. (2020). Plasma Levels of CGRP and Expression of Specific microRNAs in Blood Cells of Episodic and Chronic Migraine Subjects: Towards the Identification of a Panel of Peripheral Biomarkers of Migraine?. J. Headache Pain.

[113] Scragg J.L., Dallas M.L., Wilkinson J.A., Varadi G., Peers C. (2008). Carbon Monoxide Inhibits L-Type Ca2+ Channels via Redox Modulation of Key Cysteine Residues by Mitochondrial Reactive Oxygen Species. J. Biol. Chem..

[114] Dallas M.L., Scragg J.L., Peers C. (2008). Modulation of hTREK-1 by Carbon Monoxide. Neuroreport.

[115] Wilkinson W.J., Kemp P.J. (2011). Carbon Monoxide: An Emerging Regulator of Ion Channels. J. Physiol..

[116] Dong D.-L., Zhang Y., Lin D.-H., Chen J., Patschan S., Goligorsky M.S., Nasjletti A., Yang B.-F., Wang W.-H. (2007). Carbon Monoxide Stimulates the Ca2(+)-Activated Big Conductance k Channels in Cultured Human Endothelial Cells. Hypertension.

[117] Li C.W., Ning A., Yang C., Ji R.A., Faraci A.L., Wang N. (1999). Carbon Monoxide and Cerebral Microvascular Tone in Newborn Pigs. Am. J. Physiol..

[118] Al-Karagholi M.A.-M., Gram C., Nielsen C.A.W., Ashina M. (2020). Targeting BKCa Channels in Migraine: Rationale and Perspectives. CNS Drugs.

[119] Wulf-Johansson H., Amrutkar D.V., Hay-Schmidt A., Poulsen A.N., Klaerke D.A., Olesen J., Jansen-Olesen I. (2010). Localization of Large Conductance Calcium-Activated Potassium Channels and Their Effect on Calcitonin Gene-Related Peptide Release in the Rat Trigemino-Neuronal Pathway. Neuroscience.

[120] Akerman S., Holland P.R., Lasalandra M.P., Goadsby P.J. (2010). Inhibition of Trigeminovascular Dural Nociceptive Afferents by Ca(2+)-Activated K(+) (MaxiK/BK(Ca)) Channel Opening. Pain.

[121] Bowen E.J., Schmidt T.W., Firm C.S., Russo A.F., Durham P.L. (2006). Tumor Necrosis Factor-Alpha Stimulation of Calcitonin Gene-Related Peptide Expression and Secretion from Rat Trigeminal Ganglion Neurons. J. Neurochem..

[122] Capuano A., De Corato A., Lisi L., Tringali G., Navarra P., Dello Russo C. (2009). Proinflammatory-Activated Trigeminal Satellite Cells Promote Neuronal Sensitization: Relevance for Migraine Pathology. Mol. Pain.

[123] De Corato A., Lisi L., Capuano A., Tringali G., Tramutola A., Navarra P., Dello Russo C. (2011). Trigeminal Satellite Cells Express Functional Calcitonin Gene-Related Peptide Receptors, Whose Activation Enhances Interleukin-1β pro-Inflammatory Effects. J. Neuroimmunol..

[124] Thalakoti S., Patil V.V., Damodaram S., Vause C.V., Langford L.E., Freeman S.E., Durham P.L. (2007). Neuron-Glia Signaling in Trigeminal Ganglion: Implications for Migraine Pathology. Headache.

[125] Reuter U., Bolay H., Jansen-Olesen I., Chiarugi A., Sanchez del Rio M., Letourneau R., Theoharides T.C., Waeber C., Moskowitz M.A. (2001). Delayed Inflammation in Rat Meninges: Implications for Migraine Pathophysiology. Brain J. Neurol..

[126] Reuter U., Chiarugi A., Bolay H., Moskowitz M.A. (2002). Nuclear Factor-kappaB as a Molecular Target for Migraine Therapy. Ann. Neurol..

[127] Pedersen S.H., Ramachandran R., Amrutkar D.V., Petersen S., Olesen J., Jansen-Olesen I. (2015). Mechanisms of Glyceryl Trinitrate Provoked Mast Cell Degranulation. Cephalalgia Int. J. Headache.

[128] Di Bello M.G., Berni L., Gai P., Mirabella C., Ndisang J.F., Masini E., Bani Sacchi T., Mannaioni P.F. (1998). A Regulatory Role for Carbon Monoxide in Mast Cell Function. Inflamm. Res..

[129] Takamiya R., Murakami M., Kajimura M., Goda N., Makino N., Takamiya Y., Yamaguchi T., Ishimura Y., Hozumi N., Suematsu M. (2002). Stabilization of Mast Cells by Heme Oxygenase-1: An Anti-Inflammatory Role. Am. J. Physiol. Heart Circ. Physiol..

[130] Giniatullin R. (2022). 5-Hydroxytryptamine in Migraine: The Puzzling Role of Ionotropic 5-HT3 Receptor in the Context of Established Therapeutic Effect of Metabotropic 5-HT1 Subtypes. Br. J. Pharmacol..

[131] Balcziak L.K., Russo A.F. (2022). Dural Immune Cells, CGRP, and Migraine. Front. Neurol..

[132] Moye L.S., Pradhan A.A.A. (2017). Animal Model of Chronic Migraine-Associated Pain. Curr. Protoc. Neurosci..

[133] Shelukhina I., Mikhailov N., Abushik P., Nurullin L., Nikolsky E.E., Giniatullin R. (2017). Cholinergic Nociceptive Mechanisms in Rat Meninges and Trigeminal Ganglia: Potential Implications for Migraine Pain. Front. Neurol..

[134] Mikhailov N.V., Mamontov O.A., Kamshilin A.A., Giniatullin R. (2016). Parasympathetic Cholinergic and Neuropeptide Mechanisms of Migraine. Anesthesiol. Pain Med..

